# Inhibition of Competence Development, Horizontal Gene Transfer and Virulence in *Streptococcus pneumoniae* by a Modified Competence Stimulating Peptide

**DOI:** 10.1371/journal.ppat.1002241

**Published:** 2011-09-01

**Authors:** Luchang Zhu, Gee W. Lau

**Affiliations:** Department of Pathobiology, University of Illinois at Urbana-Champaign, Urbana, Illinois, United States of America; New York Medical College, United States of America

## Abstract

Competence stimulating peptide (CSP) is a 17-amino acid peptide pheromone secreted by *Streptococcus pneumoniae*. Upon binding of CSP to its membrane-associated receptor kinase ComD, a cascade of signaling events is initiated, leading to activation of the competence regulon by the response regulator ComE. Genes encoding proteins that are involved in DNA uptake and transformation, as well as virulence, are upregulated. Previous studies have shown that disruption of key components in the competence regulon inhibits DNA transformation and attenuates virulence. Thus, synthetic analogues that competitively inhibit CSPs may serve as attractive drugs to control pneumococcal infection and to reduce horizontal gene transfer during infection. We performed amino acid substitutions on conserved amino acid residues of CSP1 in an effort to disable DNA transformation and to attenuate the virulence of *S. pneumoniae*. One of the mutated peptides, CSP1-E1A, inhibited development of competence in DNA transformation by outcompeting CSP1 in time and concentration-dependent manners. CSP1-E1A reduced the expression of pneumococcal virulence factors choline binding protein D (CbpD) and autolysin A (LytA) *in vitro*, and significantly reduced mouse mortality after lung infection. Furthermore, CSP1-E1A attenuated the acquisition of an antibiotic resistance gene and a capsule gene *in vivo*. Finally, we demonstrated that the strategy of using a peptide inhibitor is applicable to other CSP subtype, including CSP2. CSP1-E1A and CSP2-E1A were able to cross inhibit the induction of competence and DNA transformation in pneumococcal strains with incompatible ComD subtypes. These results demonstrate the applicability of generating competitive analogues of CSPs as drugs to control horizontal transfer of antibiotic resistance and virulence genes, and to attenuate virulence during infection by *S. pneumoniae*.

## Author Summary

*Streptococcus pneumoniae* is a major cause of pneumonia, ear infection and meningitis. Antibiotic resistance among *S. pneumoniae* isolates is increasingly a major clinical problem. The acquisition of antibiotic resistance genes in *S. pneumoniae* is controlled by a peptide pheromone called competence-stimulating peptide (CSP). CSP binds to a receptor called ComD, which in turn activates its cognate transcription factor ComE to initiate DNA uptake and integration into the *S. pneumoniae* genome. CSP-ComD/E also regulates the expression of virulence factors required for infection. In this study, multiple synthetic analogues of CSP pheromone were examined for their ability to inhibit acquisition of exogenous DNA, and to control infection by *S. pneumoniae* in mice. Two of these analogues, CSP1-E1A and CSP2-E1A, competitively inhibit the ability of *S. pneumoniae* to acquire the streptomycin resistance *rpsL* gene and the capsule gene *cap3A* during mouse models of acute pneumonia and bacteremia. CSP1-E1A also reduces mouse mortality during lung infection by *S. pneumoniae*. This is the first demonstration of the use of CSP analogues to attenuate virulence and to inhibit acquisition of an antibiotic resistance gene in *S. pneumoniae*. Because the CSP-ComD/E system is conserved among many pathogenic bacteria, CSP analogues may be applicable to reduce the spread of antibiotic resistance genes and to treat infections.

## Introduction

*Streptococcus pneumoniae* is one of the most important pathogens that cause bacterial pneumonia, otitis media, meningitis and sepsis [1]–[3]. The incidence of antibiotic resistance in *S. pneumoniae* has increased dramatically in the recent decades [4]–[6]. The acquisition and spread of antibiotic resistance genes in *S. pneumoniae* is at least partly due to genetic transformation, which occurs when the bacteria enter the competent state [7]–[9]. The binding of competence stimulating peptide (CSP) to its membrane-associated histidine kinase receptor ComD initiates competence in *S. pneumoniae* [7]–[10]. Upon interacting with CSP, ComD phosphorylates the cognate transcriptional regulator ComE [11]–[13]. ComE then initiates the transcription of a set of 24 genes (early genes), including *comX*. As an alternative sigma factor, ComX positively regulates the transcription of 81 genes (late genes) in the competence regulon [14]–[15]. Some of these late genes encode effectors for DNA uptake and recombination [14]. DNA sequence analysis from 60 pneumococcal isolates predicts the existence of six distinct CSP subtypes [16], [17]. However, an overwhelming majority of *S. pneumoniae* strains produce two of these subtypes: CSP1 or CSP2 [17]. Furthermore, apart from CSP1 and CSP2, the ability of other CSP subtypes to induce DNA transformation has not been analyzed. There are two major corresponding variants of ComD, named ComD1 and ComD2 [18]. CSP1 and CSP2 share 50% amino acid identity. The major sequence variation between CSP1 and CSP2 occurs in the central region of these peptides, which confers receptor specificity [19]. In contrast, the first three amino acid residues of the N-terminus and the last two amino acid residues of the C-terminus are conserved between CSP1 and CSP2. Competence in pneumococcal strains with the ComD1 receptor could be induced more efficiently with the “compatible” CSP1. In contrast, ComD2 strains are more sensitive to induction by the “compatible” CSP2 [19].

Competence for DNA transformation plays a crucial role in the ability of *S. pneumoniae* to gain virulence and antibiotic resistance genes from other species. Importantly, in recent years, the competence regulon of *S. pneumoniae* has been shown to cross regulate virulence [20]–[24]. For example, Lau *et al* [20] reported that a loss of function in ComB, an accessory protein to the ComA ABC transporter required for the export of CSP, as well as a loss of function in the ComD histidine kinase, attenuate the ability of *S. pneumoniae* to cause pneumonia and bacteremia in mice. This report has been subsequently confirmed by other studies [21]–[23]. In addition, it has been shown that competence-mediated cell lysis may mediate the release of pneumolysin, as well as the cell wall component lipoteichoic acid (LTA), the former being an important virulence factor of *S. pneumoniae* [23], [24]. Microarray analysis has indicated that CSP induces the transcription of both virulence and stress responsive genes [14]. These above-mentioned studies indicate that activation of the competence regulon is not only essential for DNA uptake and transformation, but also important for virulence. Therefore, the competence regulon represents an attractive drug target to combat both pneumococcal infections and the spread of antibiotic resistance genes.

In this study, we performed amino acid substitutions in CSP1 and CSP2, and examined the ability of these CSP variants to inhibit the development of competence, horizontal gene transfer, and virulence of *S. pneumoniae* both *in vitro* and in mice. One of these modified peptides, CSP1-E1A, was able to competitively inhibit the development of competence for DNA transformation and expression of virulence gene *in vitro*. Importantly, CSP1-E1A was able to attenuate the virulence of *S. pneumoniae* during lung infection in mice, as well as inhibiting the ability of pneumococcus to acquire both an antibiotic resistance gene and a capsule gene during mouse models of acute pneumonia and bacteremia infections. Significantly, we demonstrated that the same amino acid substitution on CSP2 (CSP2-E1A) also inhibits CSP2-mediated competence development. Moreover, both CSP1-E1A and CSP2-E1A were capable of cross inhibiting the induction of competence regulon mediated by incompatible receptors, ComD2 and ComD1, respectively.

## Results

### The first and the third amino acid residues on the N terminus of CSP1 are critical for the induction of competence

**Figure 1:**
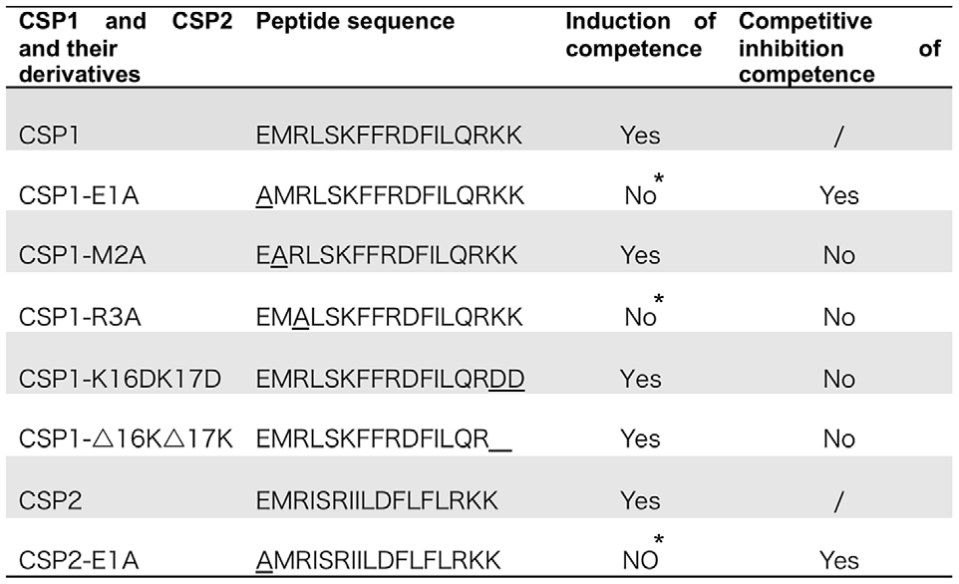
Amino acid sequences of CSP1 and CSP2, and their analogues. Modified CSPs were synthesized based on the sequence of CSP1 or CSP2 by amino acid substitutions or by deletion. The ability of each CSP analogue to induce competence and to competitively inhibit CSP1 or CSP2-mediated competence is presented. *No represents < 1% of CSP1 or CSP2 activity during the induction of competence.

**Figure 2:**
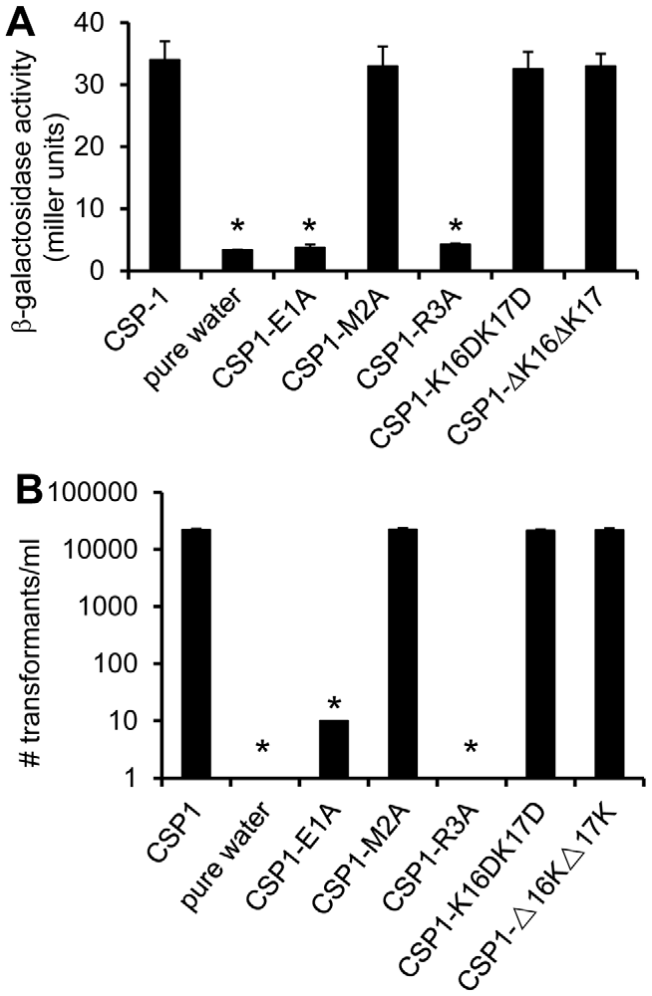
The first and third amino acid residues of CSP1 are essential for induction of competence. (A) D39pcomX::lacZ cells were incubated with CSP1 or with individual modified peptides at a final concentration of 100 ng/ml for 30 min. The ability of each peptide to induce competence regulon was measured by induction of the *comX* promoter in D39pcomX::lacZ. The amino acid substitution of 1^st^ amino acid (CSP1-E1A) and 3^rd^ amino acid (CSP1-R3A) in the N-terminus abolished the peptide's ability to induce the expression of *comX*. (B) D39 cells were exposed to 100 ng/ml of CSP1 or individual modified peptides. DNA transformation was performed using the *rpsL* gene. Transformants were selected on THB agar containing 100 µg/ml streptomycin. Experiments were performed in triplicates and repeated three times. The means ± SD of one typical experiment are shown. *p<0.01 when compared against CSP1-treated cells.

Because amino acid residues in the central region of CSP1 are important for receptor specificity [19], we hypothesized that conserved amino acid residues on both the N-terminus and the C-terminus of CSP1 are important for its ability to induce competence. By amino acid substitutions or deletions of the first three amino acid residues on the N-terminus and of the last two amino acids on the C-terminus, we synthesized five CSP1 variants (Figure 1). The ability of these modified peptides to induce competence in *S. pneumoniae* wild-type strain D39 (Table 1) grown in Todd Hewitt Broth (THB) was compared to that of CSP1 by: (i) monitoring the transformation frequency of a *rpsL* gene that confers resistance to streptomycin [25], and (ii) monitoring the promoter activity of the competence specific sigma factor gene *comX*. Induction of ComX indicates that *S. pneumoniae* cells have entered a competent state. The promoter activity of *comX* was monitored by assaying the ß-galactosidase activity in D39pcomX::lacZ, a D39 derivative with a *lacZ* gene fused behind the *comX* promoter (Table 1) [26]. Deletion or substitution of either of the two lysine (K) residues on the C-terminus (CSP1-K16DK17D, CSP1-ΔK16ΔK17) did not alter the ability of these peptides to induce competence (Fig. 2A–2B). In contrast, substitution of the first amino acid (glutamate to alanine) (CSP1-E1A) severely reduced the ability of this peptide to induce *comX* (Fig. 2A) and DNA transformation (Fig. 2B). CSP1-E1A could only induce about 1% of *comX* promoter activity and > 3 logs lower DNA transformation when compared to CSP1. Similarly, alanine substitution of the third residue of the N terminus (CSP1-R3A) impaired its ability to induce both *comX* and DNA transformation (Fig. 2A–2B). In contrast, alanine substitution of the second amino acid residue on the N terminus (CSP1-M2A) did not alter its ability to induce both *comX* and DNA transformation (Fig. 2A–2B).

**Table 1:** *S. pneumoniae* strains used in this study.

Strains	Relevant characteristics	Reference
D39	Wild-type (CSP1, ComD1)	[49]
D39pcomX::lacZ	D39 with a promoterless *lacZ* reporter gene behind *comX* promoter	This work
D39pcbpD::lacZ cells	D39 with a promoterless *lacZ* reporter gene behind *cbpD* promoter	This work
TIGR4	Wild-type (CSP2, ComD2)	[51]
TIGR4pcomX::lacZ	TIGR4 with a promoterless *lacZ* reporter gene behind *comX* promoter	This work
R6	A derivative of D39 without capsule	[50]
CPM3	R6 with a promoterless *lacZ* reporter gene behind *comX* promoter	[26]
CP1298	R6 strain with a modified *rpsL* gene that confers resistance to streptomycin	[27]
0100993	Wild-type, serotype 3	[20]
*cap3A*	A non-encapsulated 0100993 derivative generated by deleting *cap3A* gene using Janus cassette	This work

### CSP1-E1A but not CSP1-R3A out-competes and inhibits CSP1-mediated *comX* induction in a dose and time dependent manner

**Figure 3:**
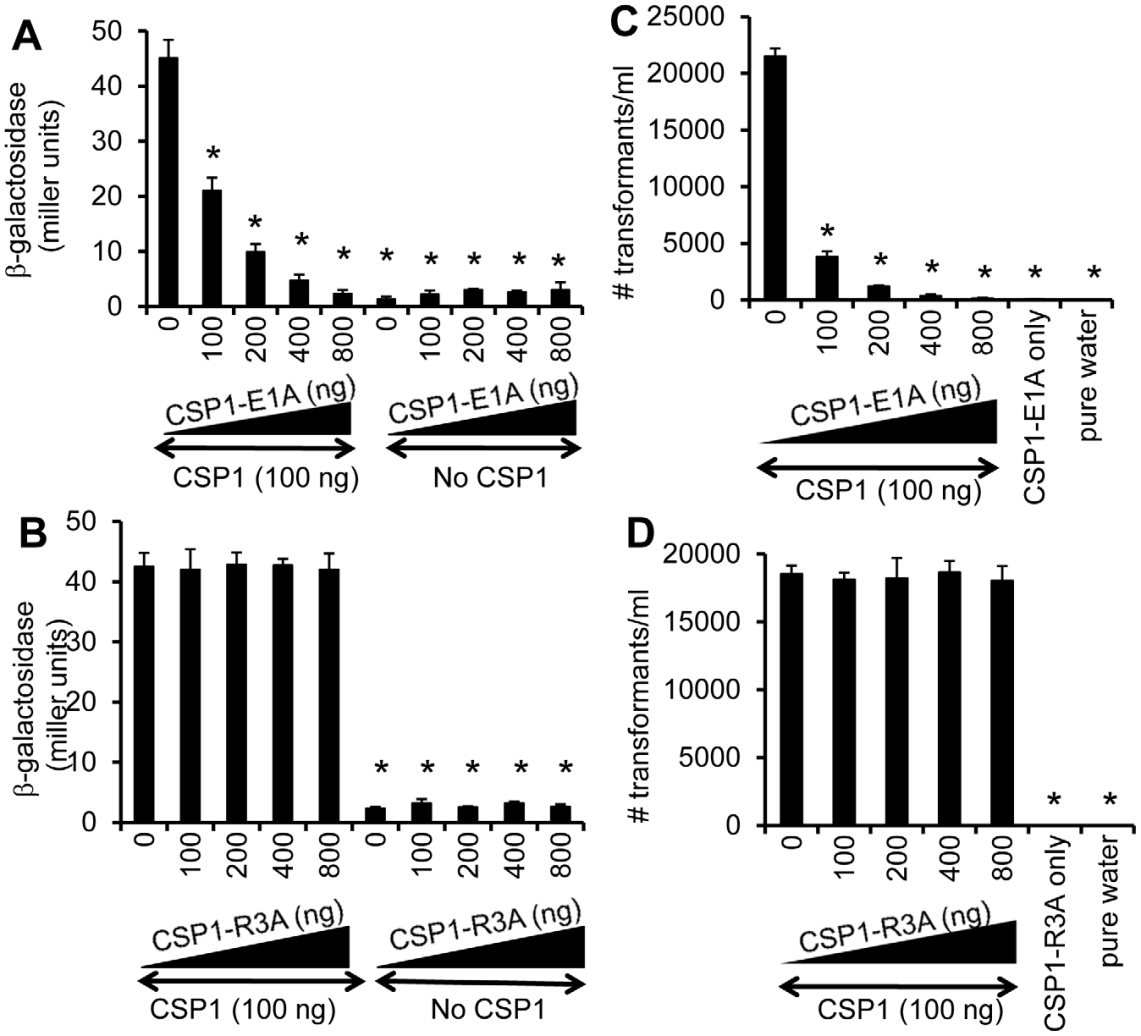
Dose dependent inhibition of *comX* expression and DNA transformation by CSP-E1A. (A–B) CSP-E1A but not CSP1-R3A inhibits CSP1-mediated induction of *comX* in a dose dependent manner. D39pcomX::lacZ cells were exposed to 100 ng/ml of CSP1 alone or simultaneously with increasing concentrations of CSP-E1A or CSP-R3A. The activity of *comX* gene promoter was measured by b-galactosidase activity. (A) CSP-E1A competitively inhibits the ability of CSP1 to induce *comX*. (B) CSP-R3A is unable to induce *comX*, and does not competitively inhibit CSP1. (C–D) CSP-E1A but not CSP1-R3A outcompetes and inhibits CSP1-mediated genetic transformation in a dose dependent manner. *In vitro* transformation was performed by treating D39 with 100 ng/ml CSP1 alone or simultaneously with increasing concentrations of CSP1-E1A or CSP1-R3A. Purified PCR product harboring a mutated *rpsL* gene was used for genetic transformation. Transformants were selected on THB agar plates supplemented with 100 µg/ml streptomycin. CSP1-E1A competitively inhibited the ability of CSP1 to induce DNA transformation (C). CSP1-R3A was unable to competitively inhibit DNA transformation (D). Experiments were performed in triplicates and repeated three times. The means ± SD of one typical experiment are shown. *p<0.01 when compared against cells treated with CSP1 only.

As presented in Figure 2, two modified CSP peptides—CSP-E1A and CSP-R3A—are attenuated in their ability to induce the *comX* gene and the competence of DNA transformation. We examined the ability of these two peptides to competitively inhibit the induction of competence by CSP1. D39pcomX::lacZ cells were exposed to 100 ng of CSP1 alone or simultaneously to increasing amounts of CSP1-E1A (Fig. 3A) or CSP1-R3A (Fig. 3B). In the absence of CSP1-E1A, CSP1 induced the promoter activity of *comX* gene to approximately 45 Miller units of b-galactosidase. However, in the presence of CSP1-E1A, the ability of CSP1 to induce *comX* (Fig. 3A) was severely inhibited in a concentration dependent manner. CSP1-E1A by itself only induced low expression of *comX*. In contrast, even though CSP1-R3A by itself was unable to induce the expression of *comX*, it did not competitively inhibit CSP1-dependent *comX* expression (Fig. 3B). These results suggest that CSP1-E1A has the unique ability to competitively inhibit the ability of CSP1 to induce competence.

### CSP-E1A but not CSP1-R3A inhibits CSP1-mediated genetic transformation

Because of their inability to induce competence and DNA transformation, we next examined whether CSP1-E1A and CSP1-R3A were able to competitively inhibit CSP1-mediated DNA transformation. *In vitro* transformation with the *rpsL* gene was performed with *S. pneumoniae* D39 cells exposed to 100 ng of CSP1 alone or simultaneously in the presence of increasing amounts of CSP1-E1A or CSP1-R3A. As expected, in the absence of CSP1-E1A, CSP1 induced DNA uptake and transformation at high frequency (Fig. 3C). However, the presence of increasing concentrations of CSP1-E1A drastically inhibited the transformation of D39. For example, at equal concentration of 100 ng/ml, CSP1-E1A reduced CSP1-mediated transformation by 5.6-fold. Interestingly, increasing concentration of CSP1-R3A failed to competitively inhibit the ability of CSP1 to induce DNA transformation in D39 cells (Fig. 3D). These results are consistent with the inability of CSP1-R3A to inhibit the induction of *comX* by CSP1 (Fig. 3B). Collectively, these data suggest that CSP1-E1A is uniquely capable of competitively inhibiting CSP1-mediated competence induction and DNA transformation.

### CSP1-E1A inhibits the competence regulon in a time-dependent manner

**Figure 4:**
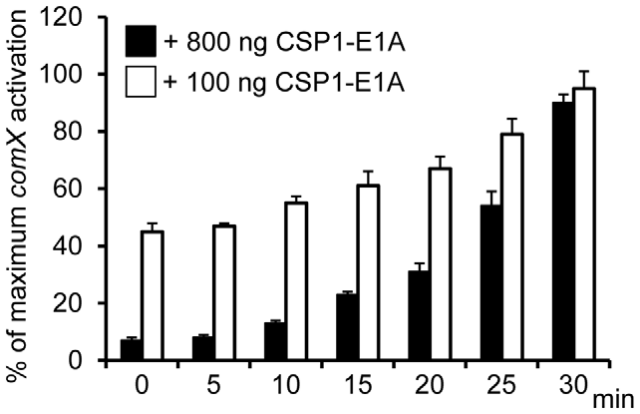
Time-dependent inhibition of CSP1 by CSP1-E1A. CSP1-E1A competitively inhibits CSP1 in a time-dependent manner. D39pcomX::lacZ cells (OD 600_nm_ 0.1) were pre-exposed to 100 ng/ml CSP1. At Time 0, 5, 10, 15, 20, 25 and 30 min, CSP-E1A (100 ng or 800 ng) was added to chase CSP1. Cells were collected 30 min after each addition of CSP1-E1A for b-galactosidase assays. Activation of *comX* promoter was calculated against D39pcomX::lacZ cells exposed to CSP1 alone (100%). Experiments were performed in triplicates and repeated three times. The means ± SD of one typical experiment are shown.

Because CSP1-E1A is capable of competitively inhibiting CSP1-mediated competence induction in *S. pneumoniae*, we examined whether this inhibition occurred in a time-dependent manner. D39pcomX::lacZ cells were pre-exposed to 100 ng/ml CSP1, then chased by the addition of CSP1-E1A at indicated time intervals. Activation of the *comX* promoter was calculated against D39pcomX::lacZ cells exposed to CSP1 alone (100%). As shown in Figure 4, CSP1-E1A at concentration of 100 ng/ml was able to inhibit approximately 40–50% of *comX* induction 10 minutes after pre-induction with CSP1. The inhibition was abolished at approximately 30 minutes after preinduction with CSP1. At the concentration of 800 ng/ml, CSP1-E1A was highly effective, and able to inhibit 50% of CSP1-mediated *comX* induction 25 min after pre-induction by CSP1. These results suggest that under our *in vitro* experimental conditions, 30 min was roughly the time span required for the distribution and binding of CSP1 to ComD to prevent competitive inhibition by CSP1-E1A. In addition, these results also indicate that 30 minutes was approximately the amount of time required for establishment of full competence when *S. pneumoniae* D39 cells are exposed to CSP1.

### CSP1-E1A delays and inhibits the development of spontaneous competence *in vitro* in a concentration dependent manner

**Figure 5:**
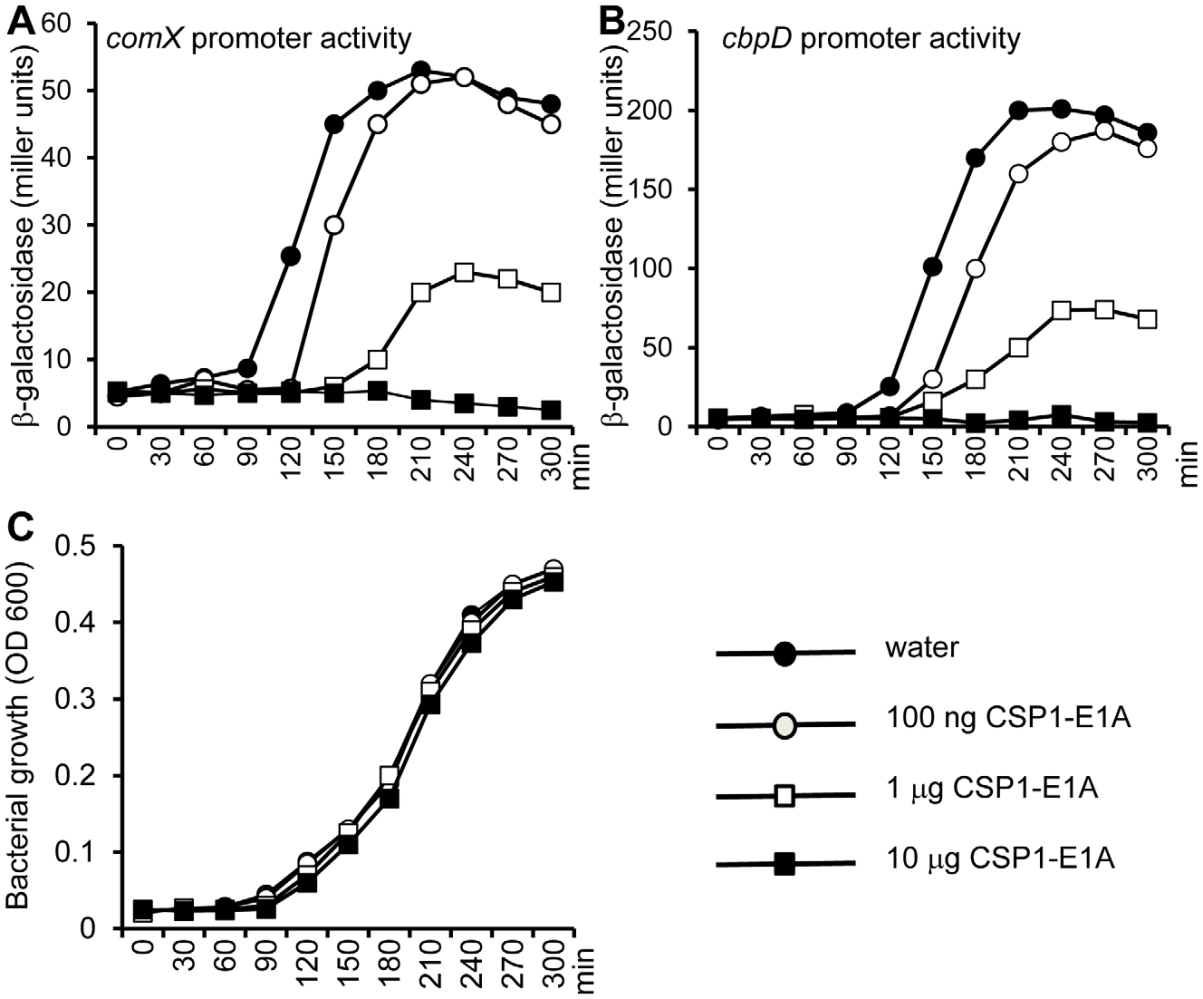
CSP1-E1A delays and inhibits the spontaneous development of competence in *S. pneumoniae* in a concentration-dependent manner. (A–B) D39pcomX::lacZ and D39pcbpD::lacZ cells grown in THB (pH 8.3) were incubated with 100 ng/ml, 1 µg/ml or 10 µg/ml of CSP1-E1A or with same volume of sterile water, and monitored for *comX* promoter activity (A) or for the *cbpD* promoter activity (B) by b-galactosidase assays at indicated time intervals. (C) CSP1-E1A does not inhibit the growth of *S. pneumoniae*. Similar results were obtained from three independent experiments. The data from one typical experiment are shown.

During its normal growth, *S. pneumoniae* cells spontaneously enter a developmental stage of competence for DNA uptake and transformation by activating the biosynthesis of CSP [27]. Because CSP1-E1A competitively inhibits the competence development in pneumococcal cells exposed to exogenously supplied CSP1, we further investigated its ability to suppress spontaneous development of competence. D39pcomX::lacZ cells grown in THB (pH 8.3) were exposed to sterile water (control) or to CSP1-E1A. The promoter activity of *comX* began to increase dramatically in the D39pcomX::lacZ cells incubated with sterile water at approximately 90 min (Fig. 5A). In contrast, in the D39pcomX::lacZ cells treated with 100 ng or 1 µg of CSP1-E1A, the induction of *comX* promoter was delayed for 30 min and 60 min, respectively (Fig. 5A). Furthermore, the maximum level *comX* promoter activity was only 38% in cells treated with 1 µg of CSP1-E1A when compared to those cells treated with sterile water. CSP1-E1A at the concentration of 10 µg/ml completely abolished the induction of *comX* promoter in D39 (Fig. 5A). Similarly, CSP1-E1A delayed and inhibited the promoter activity of a virulence gene *cbpD*, a late gene in the competence regulatory circuit, in a concentration dependent manner (Fig. 5B). CSP1-E1A did not interfere with the growth D39 cells (Fig. 5C). These results suggest that CSP1-E1A has the ability to delay and inhibit spontaneous competence development during normal growth in *S. pneumoniae* in a concentration-dependent manner.

### CSP1-E1A attenuates the expression of *S. pneumoniae* virulence factor *in vitro*

**Figure 6:**
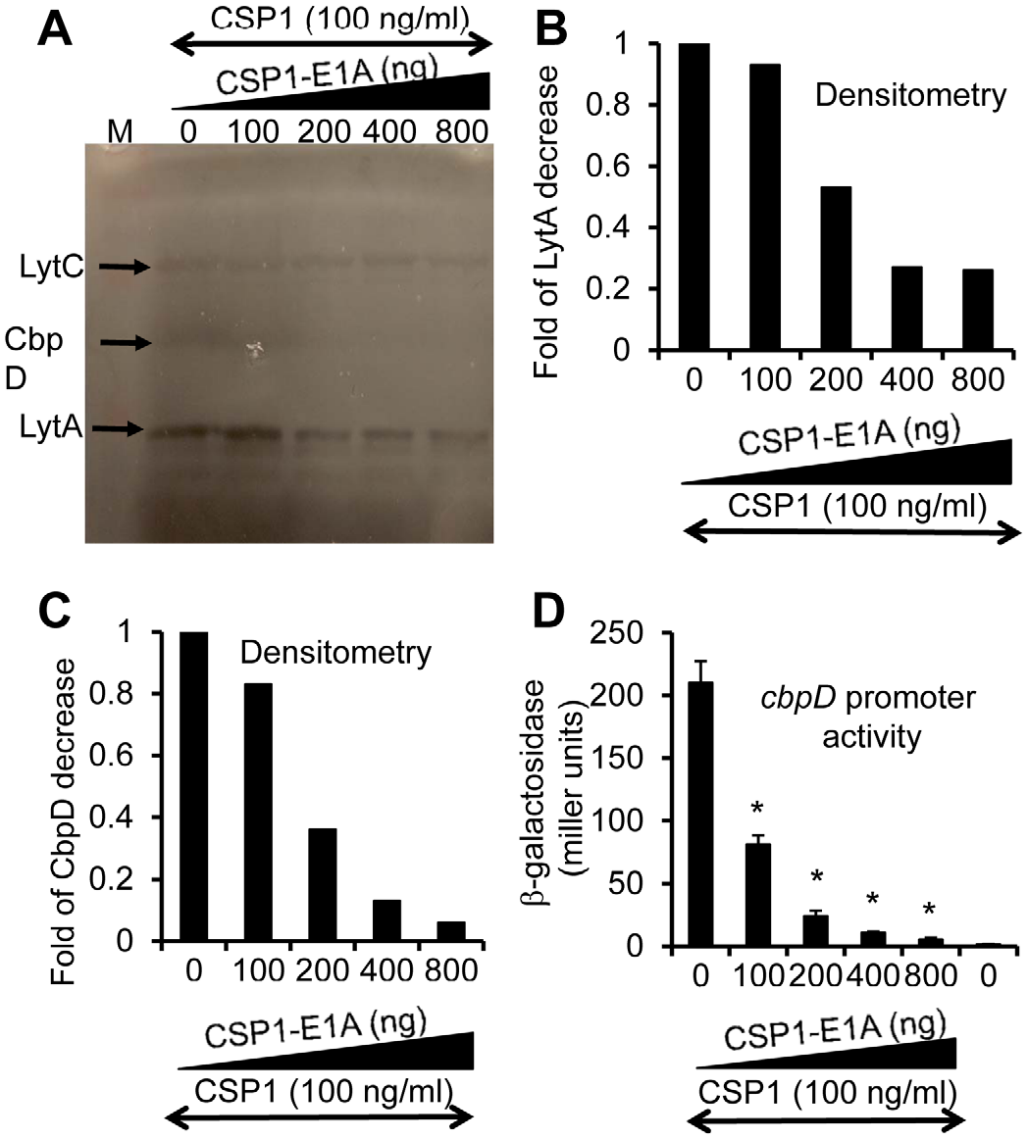
CSP1-E1A inhibits the expression of competence regulated virulence factors LytA and CbpD *in vitro*. (A) CSP1-E1A attenuates the expression of LytA and CbpD. D39 cells (OD 600_nm_ 0.1) were exposed to CSP1 (100 ng/ml) alone or simultaneously to increasing amounts of CSP1-E1A (0, 100, 200, 400 or 800 ng/ml). Cells were incubated for 30 minutes, lysed and subjected to zymogram analysis. Lytic activity was observed as bands of clear zones in the turbid gel. Experiments were repeated three times. The gel from one typical experiment is shown. (B–C) Densitometry analysis of LytA (B) and CbpD (C) from the zymogram in A. (D) CSP1-E1A inhibits the promoter activity of *cbpD* in a concentration dependent manner. D39pcbpD::lacZ cells were exposed to CSP1 alone, pure water alone or to a mixture of 100 ng/ml CSP1 and CSP-E1A.Cells were collected for b-galacosidase assays 30 min after exposure.

We and others have previously shown that the competence regulon regulates both DNA transformation and virulence [20]–[24], although the mechanism behind this regulation is not fully understood. Guiral *et al* [23] have proposed a possible connection between competence induced cell lysis and virulence. Induction of competence triggers the expression of the murein hydrolases autolysin A (LytA) and Choline-Binding Protein D (CbpD). Together, they induce pneumococcal cell lysis and the release of virulence factors including penumolysin and cell wall component LTA into the hosts. Therefore, we examined whether CSP1-E1A could inhibit the synthesis and the release of pneumococcal murein hydrolases. *S. pneumoniae* D39 cells were treated with CSP1 (100 ng/ml) alone or simultaneously with increasing amounts of CSP1-E1A (0, 100, 200, 400 or 800 ng/ml), and subjected to zymogram analysis. As shown in Figures 6A–6C, The expression of CbpD and LytA decreased by 63% and 44% respectively when D39 cells were simultaneously treated with a combination of 100 ng/ml CSP1 and 200 ng/ml CSP1-E1A. The decrease is even more profound as the concentrations of CSP1-E1A was increased to 400 ng/ml or 800 g/ml, reducing the expression of LytA by 73% and 72% (Fig. 6B). Similarly, at the concentrations of 400 ng/ml or 800 g/ml, CSP1-E1A reduced the expression of CbpD by 87% and 94%, respectively (Fig. 6C). Inhibition of *cpbD* promoter activity (Fig. 6D) mirrored the zymogram analysis. These results suggest that CSP1-E1A is able to inhibit the expression of virulence genes regulated by competence regulon.

### CSP1-E1A attenuates the virulence of *S. pneumoniae* during infection of mouse lungs

**Figure 7:**
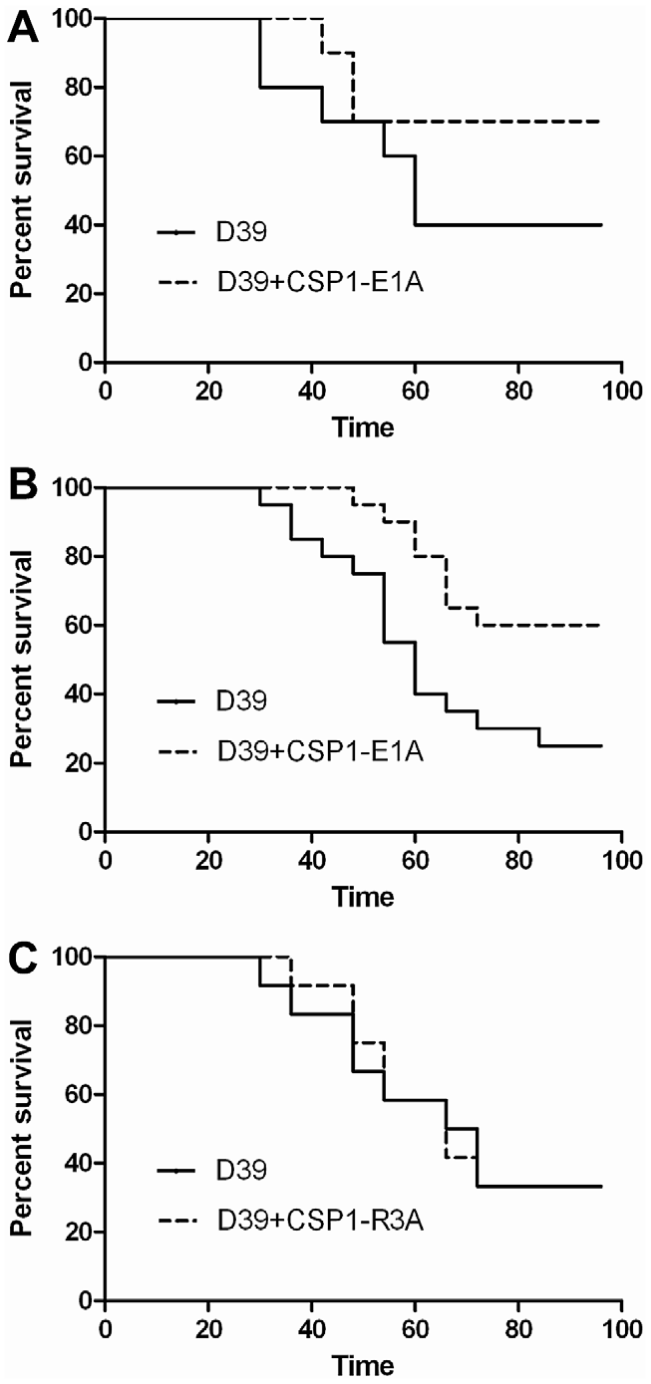
CSP1-E1A attenuates the mortality of mice infected with *S. pneumoniae*. (A) Six-week old CD-1 mice (n = 10) were intranasally infected with *S. pneumoniae* D39 (3×10^6^, 50 µl) alone, or with a mixture of D39-CSP-E1A (20 µg/mouse). Infected mice were monitored every 6 hr. Moribund mice were euthanized and considered dead. The difference in kinetics of mouse survival between the two groups was not statistically significant (*p* = 0.21) as analyzed by the log rank (Mantel-Cox) survival analysis. (B) Infection assay was repeated with CD-1 mice (n = 20) using D39 (2×10^6^, 50 µl) alone or with a mixture of D39-CSP-E1A (100 µg/mouse). Infected mice were monitored as described in (A). The kinetics of death was significantly delayed (*p* = 0.01) as determined by the log rank (Mantel-Cox) survival analysis. (C) The survival kinetics was indistinguishable between mice infected with D39 alone or in mice infected with a mixture of D39 and 100 µg CSP1-R3A (*p* = 0.96).

The ability of CSP1-E1A to inhibit the expression of LytA and CbpD *in vitro* reveals the possibility of using this peptide analogue to attenuate pneumococcal infection. We examined whether CSP1-E1A could attenuate the virulence of *S. pneumoniae* D39 using mouse model of acute pneumonia in two independent experiments. In Experiment 1, adult CD1 mice (groups of 10) were intranasally infected with D39 alone or a mixture of D39 and 20 µg CSP1-E1A. Animals were monitored closely, and those that were moribund were considered dead. As shown in Figure 7A, inclusion of CSP1-E1A in the infection mix significantly reduced the mortality rate of infected mice from 60% (D39 alone) to 30% (D39 coinoculated with CSP1-E1A). However, the difference in kinetics of mouse survival between the two groups was not statistically significant (*p* = 0.21) as analyzed by the log rank (Mantel-Cox) survival analysis. Thus, we repeated the experiment with higher number of mice and increased concentration of CSP1-E1A. In Experiment 2, CD1 mice (groups of 20) were intranasally infected with D39 alone or a mixture of D39 and 100 µg CSP1-E1A. As shown in Figure 7B, increasing the CSP1-E1A concentration to 100 µg/mouse in the infection mix significantly reduced the mortality rate of infected mice from 75% (D39 alone) to 40% (D39 coinoculated with CSP1-E1A). In addition, the kinetics of death was delayed, and was statistically significant (*p* = 0.01) (Fig. 7B). In contrast, the survival rate and mortality kinetics were indistinguishable between mice infected with D39 alone or in mice infected with a mixture of D39 and 100 µg CSP1-R3A (Fig. 7C) (*p* = 0.96). These results suggest that CSP1-E1A is uniquely able to attenuate virulence mechanisms regulated by the competence regulon during *S. pneumoniae*-mediated lung infections.

### CSP1-E1A inhibits the horizontal gene transfer *in vivo*

The competence regulon plays a role in the acquisition and spread of antibiotic resistant genes in *S. pneumoniae* [7]–[9]. *In vivo* gene uptake and transformation of *S. pneumoniae* have been previously reported to occur at extremely low frequency [28]–[31]. As we have shown above, CSP1-E1A has the ability to competitively inhibit CSP1-mediated DNA transformation, and attenuate the development of spontaneous competence in *S. pneumoniae in vitro*. Here, we examined whether CSP-E1A could inhibit acquisition of the streptomycin resistance *rpsL* gene *in vivo,* using mouse models of acute pneumonia and bacteremia that we had previously described [20]. Adult CD1 mice were intranasally or intraperitoneally infected with a transformation mixture composed of D39 bacteria and *rpsL* donor DNA and CSP1, or a transformation mixture composed of D39 bacteria and *rpsL* donor DNA and CSP1 and CSP1-E1A. To ensure that no transformation occurred outside animals, each component of the transformation mixture was kept on ice. Individual transformation components were mixed on ice and immediately inoculated into the animals. Plating of the ice-cold transformation mixture before infection or at time intervals 10, 20 and 30 min after infection did not give rise to any transformants (data not shown). Intranasally-infected mice were incubated for 6 hr whereas intraperitoneally-infected mice were incubated until moribund (∼16–24 hr). As shown in Table 2, all five mice intranasally-infected with the D39-rpsL DNA-CSP1 mixture gave rise to a range of 3 to 41 streptomycin resistant transformants out of the total lung output of 10^6^–10^7^ D39 cells. In contrast, no streptomycin resistant D39 was recovered in the five mice infected with D39-*rpsL* DNA-CSP1-CSP1-E1A mixture. Similarly as shown in Table 3, all four mice intraperitoneally-infected with the D39-rpsL DNA-CSP1 mixture gave rise to a range of 273 to 517 streptomycin resistant transformants out of the total 10^8^ D39 cells. In contrast, no streptomycin resistant D39 cells were recovered in the four mice intraperitoneally-infected with D39-*rpsL* DNA-CSP1-CSP1-E1A. Collectively, these results suggest that CSP1-E1A is efficient in suppressing the ability of CSP1 to activate competence regulon *in vivo*.

**Table 2:** CSPP1-E1A inhibits the ability of *S. pneumoniae* to acquire the *rpsL* streptomycin resistance gene in mouse lungs during acute pneumonia.

Treatment	Mice	# of output bacteria/lung	# transformants
**CSP1**	1	5.7×10^6^	3
	2	3.3×10^7^	15
	3	7.2×10^6^	7
	4	1.4×10^7^	23
	5	2.3×10^7^	41
**CSP1 + CSP1-E1A**	1	5.3×10^6^	0
	2	1.7×10^7^	0
	3	2.1×10^7^	0
	4	3.2×10^7^	0
	5	1.2×10^7^	0

Mice (n = 5) were intranasally inoculated with D39 (3×10^7^, 50 µl volume) supplemented with 100 ng CSP1 and 10 µg of *rpsL* gene DNA, or D39 supplemented with a mixture 100 ng CSP1, 800 ng CSP1-E1A and 10 µg of *rpsL* gene DNA in THB. Mouse lungs were harvested 6 hr post-inoculation, homogenized and plated on THB agar for total bacterial load or THB agar supplemented with streptomycin (100 µg/ml) for *rpsL* transformants.

**Table 3:** CSPP1-E1A inhibits the ability of *S. pneumoniae* to acquire the *rpsL* streptomycin resistance gene in mouse peritoneal cavity.

Treatment	Mice	# of output bacteria/spleen	# transformants
**CSP1**	1	3.1×10^8^	273
	2	4.5×10^8^	251
	3	2.7×10^8^	372
	4	5.7×10^8^	517
**CSP1 + CSP1-E1A**	1	1.3×10^8^	0
	2	4.3×10^8^	0
	3	2.2×10^8^	0
	4	2.4×10^8^	0

Mice were intraperitoneally infected with D39 (3×10^7^, 50 µl volume) supplemented with 100 ng CSP1 and 10 µg of *rpsL* gene DNA, or D39 supplemented with a mixture 100 ng CSP1, 800 ng CSP1-E1A and 10 µg of *rpsL* gene DNA in THB. Mouse spleens were harvested between 16–24 hr post-infection, homogenized and plated on THB agar for total bacterial load or THB agar supplemented with streptomycin (100 µg/ml) for *rpsL* transformants.

Spontaneous transformation of encapsulated *S. pneumoniae* strains including D39 occurs at extremely low frequency *in vivo*. Thus, CSP1 was included to increase the efficiency of DNA transformation experiments reported in Tables 2 and 3. Inclusion of CSP1 beckoned the question of whether CSP1-E1A could attenuate natural transformation events *in vivo*. Natural transformation experiments were performed using a capsule mutant *cap3A* (Table 1) derived from a serotype 3 isolate 0100993 [20]. The colonies of serotype 3 *S. pneumoniae* isolates are large and mucoid, and can be easily distinguished from small colonies formed by non-encapsulated rough mutants. Mice were intraperitoneally inoculated with a mixture of 3×10^9^ *cap3A* cells and 100 µg of chromosomal DNA from 0100993, or with a mixture of 3×10^9^ *cap3A* cells and 100 µg of 0100993 chromosomal DNA and 100 µg of CSP1-E1A. Higher amount of CSP1-E1A was used because we did not know the length of the inhibitory effect of this peptide analogue *in vivo*. In addition, the timing of *S. pneumoniae* cells entering the competent state for DNA transformation *in vivo* was also not clear. Transformation mixtures were assembled under ice-cold condition as described for Tables 2 and 3. Plating of transformation mixtures immediately before and after inoculation did not recover any transformants (data not shown). As shown in Table 4, transformants with wild-type capsule were recovered from four of the five mice inoculated with the mixture of *cap3A* bacteria and 0100993 genomic DNA. In contrast, transformants with wild-type capsule were only recovered from one out of five mice co-inoculated with *cap3A* bacteria and 0100993 DNA and CSP1-E1A. Furthermore, we used CSP1-R3A (Fig. 1) as a control to demonstrate the specificity of CSP1-E1A inhibition. CSP1-R3A was unable to inhibit development of natural competence *in vivo* (Table 4). In the group treated with a mixture of 3×10^9^ *cap3A* cells and 100 µg of 0100993 chromosomal DNA and 100 µg of CSP1-R3A, transformants were isolated from all the 5 mice, indicating high concentration of CSP1-R3A was unable to inhibit natural DNA transformation *in vivo*. These results suggest that CSP1-E1A has the unique ability to reduce the incidence of natural DNA transformation process *in vivo*.

**Table 4:** CSPP1-E1A attenuates the ability of *cap3A* mutant *S. pneumoniae* to acquire capsule gene during natural transformation in mouse peritoneal cavity.

Treatment	Mice	# of transformants/spleen
***cap3A*** ** bacteria + 0100993 chromosomal DNA**	1	4.7×10^6^
	2	2.5×10^6^
	3	2.0×10^7^
	4	7.7×10^6^
	5	0
***cap3A*** ** bacteria + 0100993 chromosomal DNA + CSP1-E1A**	1	0
	2	0
	3	0
	4	3.3×10^6^
	5	0
***cap3A*** ** bacteria + 0100993 chromosomal DNA + CSP1-R3A**	1	7.3×10^5^
	2	4.2×10^6^
	3	2.7×10^5^
	4	1.1×10^7^
	5	5.4×10^6^

Mice were intraperitoneally infected with *S. pneumoniae cap3A* mutant bacteria (3×10^9^, 100 µl volume) supplemented with 100 µg of chromosomal DNA from the parental wild-type strain 0100993 in the presence or absence of 100 µg CSP1-E1A or CSP1-R3A. Mouse spleens were harvested at 24 hr post-infection, homogenized, serially diluted and plated on THB agar for enumeration of mucoid colonies.

### CSP1-E1A is not bacteriostatic against *S. pnuemoniae*

A previous study has shown that CSP2 has bacteriostatic activity against *S. pneumoniae* [32]. To rule out non-specific effects due to a much higher concentration of CSP1-E1A used in the virulence attenuation (Fig. 7B) and *in vivo* natural transformation (Table 4) studies, we examined whether CSP1-E1A was bacteriostatic against *S. pneumoniae*. As shown in Figure S1A, CSP1 at concentrations of 20 µg or 100 µg caused a slight delay in the growth of D39 cells. In contrast, same concentrations of CSP1-E1A or CSP1-R3A did not slow the growth of D39 cells (Fig. S1B–S1C). These results suggest that high concentrations of CSP1-E1A is not bacteriostatic, and that attenuation of virulence and DNA transformation by CSP1-E1A is due to inhibition of competence regulon induction.

### A CSP2 analogue CSP2-E1A effectively inhibits the competence development and transformation in ComD2 strain TIGR4

**Figure 8:**
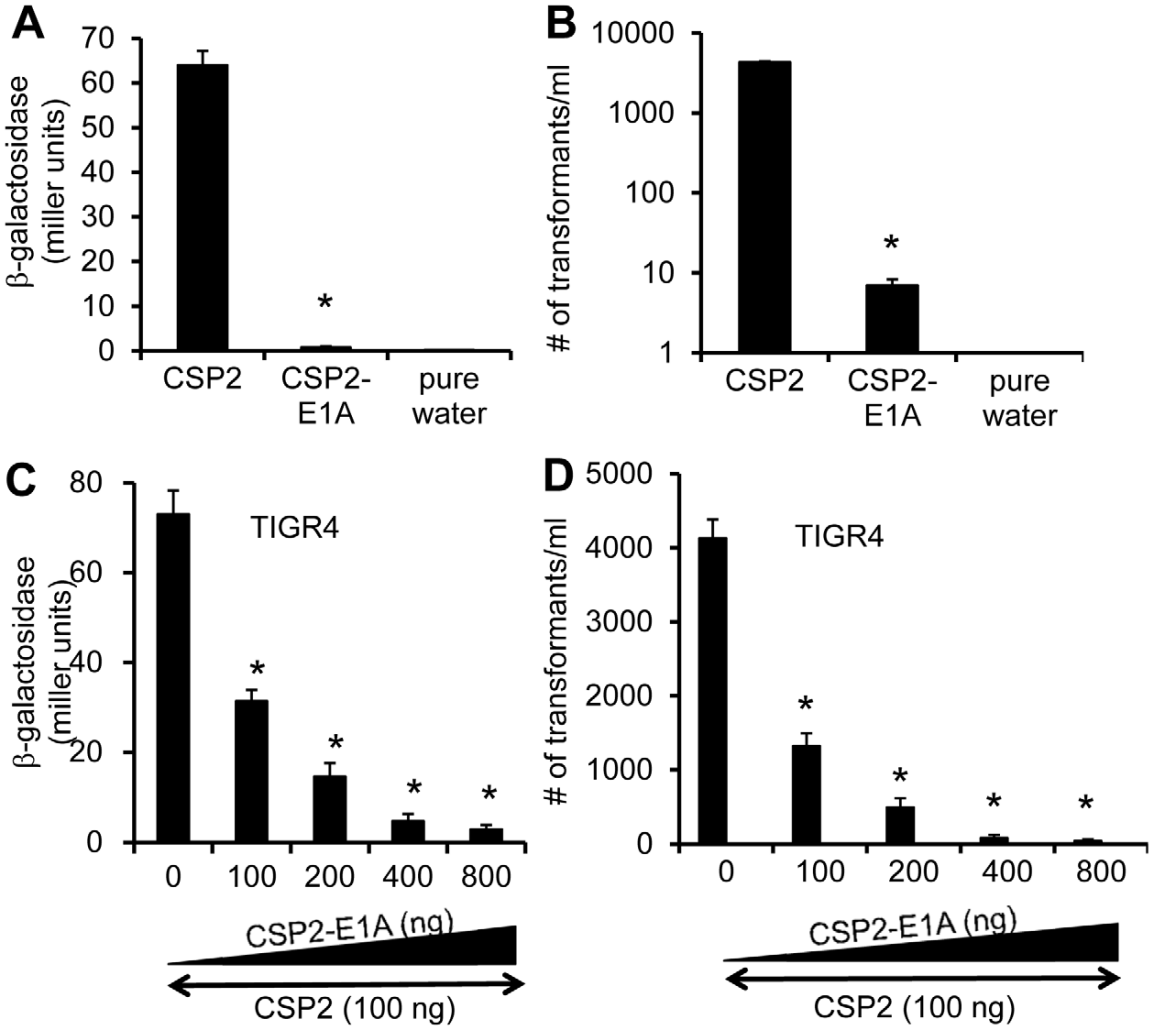
CSP2-E1A inhibits competence development and transformation in ComD2 strain TIGR4. (A–B) CSP2-E1A was drastically reduced in its ability to induce the promoter activity of comX (A) and DNA transformation in TIGR4 (B). Experiments were performed in triplicates and repeated three times. The means ± SD of one typical experiment are shown. *p<0.01 when compared against TIGR4pcomX::lacZ cells (A) or TIGR4 cells (B) treated with CSP2 only. (C) CSP-E2A out-competes CSP2 and inhibits activation of the *comX* promoter activity. TIGR4pcomX::lacZ cells (OD 600_nm_ 0.1) were incubated with 100 ng/ml CSP2 alone or simultaneously with increasing amounts of CSP2-E1A. The activity of *comX* promoter was measured by b-galactosidase assays. (D) CSP-E2A inhibits CSP2-mediated DNA transformation of TIGR4. *In vitro* transformation was performed by treating TIGR4 cells with 100 ng/ml CSP2 alone or simultaneously with increasing amount of CSP2-E1A. Purified PCR product of a mutated *rpsL* gene was used for genetic transformation. Transformants were selected on THB agar supplemented with 100 µg/ml streptomycin. Experiments in were performed in triplicates and repeated three times. The means ± SD of one typical experiment are shown. *p<0.01 when compared against TIGR4pcomX::lacZ cells (A) or TIGR4 cells (B) treated with CSP2 only.

Because CSP1-E1A is an efficient inhibitor of competence induced by CSP1, we hypothesize that a synthetic CSP2 analogue with the substitution of first residue (glutamate) with alainine (CSP2-E1A) will competitively inhibit CSP2 (Fig. 1). We examined the ability of CSP2-E1A to inhibit the induction of competence by CSP2 in the compatible ComD2 strain TIGR4 (Table 1). CSP2-E1A was barely able to induce the promoter activity of *comX* (Fig. 8A), and its ability to induce DNA transformation was reduced by approximately 3 logs when compared to CSP2 (Fig. 8B). Importantly, CSP2-E1A competitively inhibited the ability of CSP2 to induce *comX* expression in the TIGR4pcomX::lacZ cells in a concentration-dependent manner (Fig. 8C). Similarly, CSP2-E1A inhibited the ability of CSP2 to induce DNA transformation of TIGR4 in a concentration dependent manner. At equal concentration of 100 ng/ml, CSP2-E1A reduced the number of transformants generated by CSP2 by 68% (Fig. 8D). At higher concentrations of 400 and 800 ng/ml, CSP2-E1A almost completely abolished CSP2-mediated DNA transformation. These results suggest that CSP2-E1A is a strong competitive inhibitor of *comX* expression and DNA transformation in ComD2 strain TIGR4.

### CSP1-E1A and CSP2-E1A cross inhibit the competence in *S. penumoniae* strains with incompatible ComD subtypes

**Figure 9:**
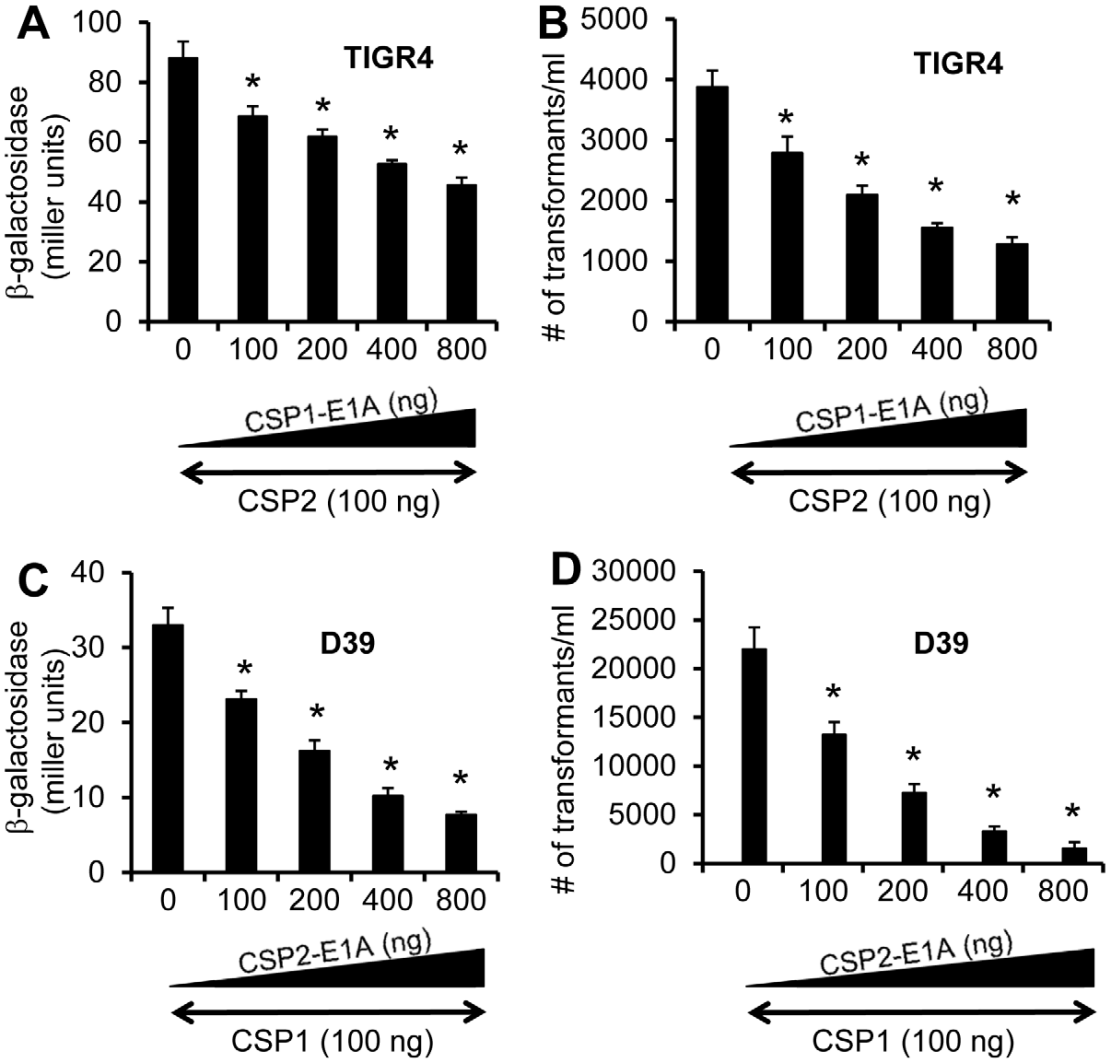
Cross inhibition of competence by CSP1-E1A and CSP2-E1A in *S. pneumoniae* strains with incompatible ComD subtypes. (A-B) TIGR4 (ComD2 subtype) or TIGR4pcomX-lacZ cells (OD 600_nm_ 0.1) were incubated with CSP2 alone or simultaneously with increasing amounts of CSP1-E1A (100–800 ng/ml). (C-D) D39 (ComD1 subtype) or D39pcomX-lacZ cells were incubated with the mixture of 100 ng/ml CSP1 alone or simultaneously with increasing amounts of CSP2-E1A (100–800 ng/ml). Pneumococcal cells were assayed for ß-galactosidase activity and transformation frequency 60 minutes after exposure. (A–B) Cross inhibition of competence in ComD2 strain TIGR4 by CSP1-E1A as measured by the induction of *comX* promoter activity (A) and transformation frequency (B). (C–D) Cross inhibition of competence in ComD1 strain D39 by CSP2-E1A as measured by the induction of *comX* promoter activity (C) and transformation frequency (D). Experiments were performed in triplicates and repeated three times. The means ± SD of one typical experiment are shown. *p<0.05 when compared against TIGR4 cells or TIGR4pcomX-lacZ cells treated with CSP2 alone, or D39 cells or D39pcomX-lacZ cells treated with CSP1 alone.

A previous study has shown that individual CSP peptide is capable of inducing the competence of DNA transformation in *S. pneumoniae* strains expressing an incompatible ComD subtype, albeit at lower efficiency [19]. For example, CSP1 is able to induce the competence at lower efficiency in TIGR4, a pneumococcal strain with an incompatible ComD2 subtype. Because the first amino acid (glutamate) at the N-terminus is conserved across all six CSP subtypes, this raises the possibility that our CSP analogues CSP1-E1A and CSP2-E1A can be broad spectrum in their ability to inhibit the development of competence, horizontal gene transfers and virulence, regardless of *S. pneumoniae* serotypes or ComD subtypes. To test this hypothesis, we examined the ability of CSP1-E1A and CSP2-E1A (Table 1) to cross inhibit the induction of *comX* and DNA transformation in ComD2 strain TIGR4 or ComD1 strain D39, respectively. As shown in Figure 9A, CSP1-E1A inhibited the induction of *comX* and DNA transformation by CSP2 in TIGR4 by a concentration dependent manner, though at a lesser efficiency. For example, CSP1-E1A at 800 ng/ml was able to inhibit approximately 50% of *comX* promoter activity in the presence of 100 ng/ml CSP2. Similarly, CSP1-E1A (800 ng/ml) decreased the CSP2-induced DNA transformation frequency by 67% (Fig. 9B).

Finally, we examined the ability of CSP2-E1A to inhibit the induction of *comX* and DNA transformation by CSP1 in the ComD1 strain D39. As shown in Figure 9C, CSP2-E1A inhibited CSP1-mediated *comX* induction and DNA transformation in D39 by a concentration dependent manner. For example, CSP2-E1A at 800 ng/ml was able to inhibit approximately 77% of *comX* induction and 90% of DNA transformation in the presence of 100 ng/ml CSP1 (Fig. 9D). Collectively, these results suggest that CSP1-E1A and CSP2-E1A have the potential of inhibiting the development of competence, horizontal gene transfer and virulence, regardless of pneumococcal serotypes and CSP/ComD subtypes.

## Discussion

The competence regulon is known to regulate DNA transformation and virulence in *S. pneumoniae* [7]–[9], [20]–[24]. In this study, we explored the use of synthetic analogues of CSPs to competitively inhibit: (i) the development of competence, (ii) horizontal gene transfer, and (iii) virulence, under both *in vitro* and *in vivo* experimental conditions. Amino acid substitution or deletion of conserved residues in C-terminus of CSP1 did not alter their ability to induce competence and DNA transformation. In contrast, amino acid substitutions on the first (CSP1-E1A) and third residue (CSP1-R3A) on the N-terminus impair the ability of these CSP analogues to induce competence. Our findings are consistent with the previously thesis work by Coomaraswamy [33], which has been summarized by Haverstein and Morrison [34]. By alanine scanning, Coomaraswamy demonstrates that both the N-terminus and the central region are more important than the C-terminus of CSP1 for the induction of competence regulon. One discrepancy of our finding from that of Coomaraswamy is on the requirement of 2^nd^ amino acid residue on the N-terminus. Coomaraswamy has shown that substituting the methionine with an alanine reduces the ability of the CSP1-M2A to induce competence by 96%. In contrast, our analysis suggests that the methionine residue is dispensable for competence induction. One possible explanation for the discrepancy is that our *comX* induction and DNA transformation experiments were performed with 100 ng/ml CSP1-M2A, approximately 10-fold higher than the amounts used by Coomaraswamy. Thus, despite its lower activity, excess amounts of CSP1-M2A might have masked our ability to see reduced efficiency of this analogue to induce competence.

Of the five CSP1 analogues examined, only CSP1-E1A is capable of competitively inhibiting the induction of competence and DNA transformation by CSP1. CSP1-E1A is able to effectively compete with CSP1 even after 25 min pre-exposure of *S. pneumoniae* to the latter. Furthermore, CSP1-E1A is able to inhibit the development of spontaneous competence in *S. pneumoniae* in a concentration dependent manner. Importantly, CSP1-E1A is able to attenuate the expression of competence-regulated pneumococcal virulence factors LytA and CbpD *in vitro,* and reduced the mortality of mice in a pneumonia model of lung infection by *S. pneumoniae*. In addition, we provide evidence that CSP1-E1A is able to inhibit the horizontal gene transfer of the streptomycin resistance *rpsL* and the capsule *cap3A* genes *in vivo.* Finally, we demonstrate a broader applicability of the peptide inhibitor strategy. CSP1-E1A and CSP2-E1A are able to cross inhibit the induction of competence and DNA transformation in pneumococcal strains with incompatible ComD subtypes. For example, CSP1-E1A is able to reduce the CSP2-mediated competence and DNA transformation in ComD2 strain TIGR4. Similarly, CSP2-E1A is able to cross inhibit the CSP1-mediated competence and DNA transformation in ComD1 strain D39.

Among the five CSP1 analogues, we found that only CSP1-E1A inhibits CSP1-mediated competence in time and concentration-dependent manners. These results suggest that glutamate, the first amino acid residue of CSPs, is only required for the activity of CSPs. Substituting the glutamate with alanine disrupts the ability of the analogue to activate ComD1, but does not interfere with its binding affinity to ComD1. Competitive inhibition of CSP1 by CSP1-E1A suggests that this analogue, which barely activates competence, can bind to the receptor kinase ComD1, and outcompete CSP1. In contrast, another CSP1 analogue, CSP1-R3A, not only loses the ability to induce competence, but also could not out compete CSP1 even when added in over-saturating concentrations. This observation indicates that CSP1-R3A has lost its ability to bind and activate ComD1. The importance of the first glutamate residue has been confirmed by our data demonstrating that the synthetic analogue CSP2-E1A also efficiently inhibits the CSP2-mediated DNA transformation in the compatible ComD2 strain TIGR4.

Several studies have revealed that the competence regulon of *S. pneumoniae* is important for virulence in mammalian hosts [20]–[24]. For example, we have previously shown that the loss-of-function mutation in the *comB* gene, which encodes an accessory protein of the ComA ABC transporter for the export of CSP, is attenuated in a mouse model of bacteremia infection [20]. Mutation of CSP receptor gene *comD* severely attenuates virulence in both mouse models of pneumonia and bacteremia infection [20]. Also, microarray analysis has showed that some virulence and stress responsive genes are upregulated during development of competence, suggesting that genes that are important for the fitness of *S. pneumoniae* are also regulated by competence CSP-ComDE cascade [14]. Thus, suppression of the CSP-ComDE cascade or downstream genes dependent on this signaling pathway may attenuate the virulence of this pathogen. Our results, which show that CSP1-E1A delays the kinetics and the rate of mouse mortality, demonstrate that this peptide has the potential to inhibit the virulence of *S. pneumoniae* by disrupting the CSP-ComDE- dependent competence regulon. Our findings are in agreement with previous reports by Oggioni *et al*, which show that administration of CSP in a mouse model of pneumonia infection increases virulence of pneumococci [35], suggesting that competence regulon is important for lung infection. In contrast, the same authors also reported that intravenous (IV) injection of CSP reduces virulence in an IV model of mouse bacteremia [32]. Based on this finding, one can surmise that administration of CSP1-E1A may have the unintended consequence of exacerbating virulence in IV model of mouse bacteremia. However, one unresolved issue with the results of the IV model of sepsis [32] is that it is in disagreement with the findings by Lau et al [20], which showed the requirement of competence regulon during pneumococcal-mediated sepsis. The discrepansy can be explained because the sepsis studies by Lau et al was performed by intraperitoneal model of infection whereas the studies by Oggionu et al was performed by IV model of infection. Neverthelss, collectively, these studies indicate the important contribution of the pneumococcal competence regulon to virulence during mouse model of pneumonia infection, and offer an tentalizing clue that the competence system as interesting drug target to combat pulmonary pneumonia. In contrast, inhibition of competence regulon by systemic delivery of CSP1-E1A in an IV model of bacteremia should be avoided until further clarification.

Within the last few decades, resistance of *S. pneumoniae* to β-lactams, macrolides, and other antibiotic classes has escalated dramatically throughout the world [4]–[6], [36]–[39]. While conjugative elements appear to be the most important causes for the spread of antibiotic resistance genes in *S. pneumoniae* [40]–[43], horizontal gene transfer of penicillin binding protein genes has occurred in clinical isolates of *S. pneumoniae* [44]. Importantly, it has been shown that under *in vitro* conditions, antibiotic stress induces genetic transformability in *S. pneumoniae* [45]. This suggests that under certain *in vivo* circumstances, instead of being killed, *S. pneumoniae* actively exploits the opportunity of antibiotic stress to acquire exogenous genes. These authors also reported that unlike wild type *S. pneumoniae*, antibiotic stress-mediated competence could not be induced in a *comA* mutant that lacks the ABC transporter needed to export CSP, suggesting that this process is CSP-dependent. Because antibiotic resistance is a serious clinical concern, the development and use of non-antibiotic based therapy to reduce horizontal gene transfer is urgently needed. Our studies suggest that CSP1-E1A and CSP2-E1A competitively inhibit CSP from binding to ComD, leading to suppression of horizontal gene transfer. The use of CSP1-E1A and CSP2-E1A, which does not inhibit the bacterial growth, is predicted to pose less selection pressure toward the survival of *S. pneumoniae*. This may alleviate the emergence of resistance in pneumococcus. More importantly, CSP1-E1A and CSP2-E1A have the ability to cross inhibit the induction of the competence regulon in *S. pneumoniae* strains with incompatible ComD subtypes. Thus, it may be beneficial to generate analogues of CSPs as drugs to reduce horizontal transfer of antibiotic resistance and virulence genes, and to attenuate virulence during infection by *S. pneumoniae* irrespective of their serotypes. In the same context, chemical inhibitors of that interfere with CSP functions may be useful as well.

In previously published studies, some of the *in vivo* transformation experiments involved infections were allowed to run their course, and success or failure of gene transfers were expressed by the number of deceased animals harboring encapsulated or antibiotic resistant transformants [28]–[31]. However, at these late stages of infection, most transformants are expected to have over-proliferated, and it may be difficult to assess the actual transformation frequency. Other transformation studies lasted for eight hr [46], [47]. In this study, we used both approaches. We assessed the infected lungs for streptomycin resistant transformants 6 hr post-infection in an effort to estimate the actual transformation frequency. Our results suggest that, despite the provision of CSP1 and abundant amounts of DNA, *in vivo* transformation events for the encapsulated wild-type *S. pneumoniae* strain D39 in mouse lungs remain low, in the range of 10^−6^ to 10^−7^ under our experimentation conditions. In comparison, we also examined the *in vivo* transformation within intraperitoneally-infected mice in which the infection was allowed to take its full course (∼ 24 hr), similar to previously published studies [28]–[31]. The percentage of transformants in D39 increases to the range of 10^−4^ to 10^−5^. The different rate of transformation may suggest that the environment in the lung is more complex and less conducive to DNA uptake and transformation than the intraperitoneal space. Alternatively, longer period of infection may have allowed more recipient D39 cells to be transformed.

Importantly, we show that CSP1-E1A is also able to attenuate natural transformation in the absence of exogenously provided CSP1. The capsule-deficient *cap3A* mutant derived from the serotype 3 strain 0100993 was used as recipient. It is well known that natural transformation frequency of capsule-deficient *S. pneumoniae* mutants is much higher than their respective encapsulated parental wild-type strains. Furthermore, recipients that have acquired the wild-type capsule gene will be more resistant to phagocytosis by host leukocytes, allowing a better possibility for the transformants to proliferate inside the host. Because of the higher transformation frequency was anticipated for the rough *cap3A* mutants, larger amounts of CSP1-E1A was used to attenuate natural transformation to ensure that the inhibition effect of CSP1-E1A could outlast and suppress spontaneous development of competence of *cap3A* cells in the peritoneal cavity. In contrast, the presence of capsule in D39 cells reduces the spontaneous transformation frequency to a very low level. Not surprisingly, 800 ng of CSP1-E1A was adequate to completely abolish transformation of D39 cells *in vivo*.

In summary, we have demonstrated the efficacy of using competitive inhibitor of CSPs to attenuate virulence and to inhibit horizontal gene transfer in *S. pneumoniae*. Because competence peptide-mediated quorum sensing systems are conserved in many pathogenic species of Gram-positive bacteria [48]–[52], the strategy of using peptide analogue may be applicable to reduce the incidence of horizontal gene transfer and acquisition of antibiotic resistance genes, and to also treat lung infections mediated by this class of pathogens. In addition, competence systems in other streptococcal species, including *S. mitis,* are very similar to that of *S. pneumoniae*, lending to the idea that the former could be transformed by pneumococcal DNA [53], [54]. *S. mitis* is generally considered as an avirulent species, but it has the potential to acquire virulence determinants from *S. pneumoniae* to transform itself into a pathogen. In this scenario, CSP1-E1A or CSP2-E1A may be applicable to reduce the emergence of the new pathogen.

## Materials and Methods

### Chemicals

All chemicals, except where noted, were purchased from Sigma Chemical Co. (St. Lois, MO).

### Bacterial strains and growth conditions

*S. pneumoniae* strains D39 [55], R6 [56] and TIGR4 [57] were generous gifts from Dr. David Briles (University of Alabama-Birmingham). Strains D39pcomX::lacZ and TIGR4pcomX::lacZ, both of which carry an insertion of the *lacZ* gene under the control of the *comX* promoter, were generated by transforming D39 and TIGR4 with the genomic DNA from CPM3 [26]. Strain D39pcbpD::lacZ was generated using previously described primers [58]. Briefly, a 500 bp fragment within *cbpD* gene was amplified and cloned into *lacZ* reporter plasmid pEVP3 [59]. The recombinant plasmid was transformed into D39 to generate D39pcbpD::lacZ. The capsule-deficient *cap3A* mutant was generated by deleting *cap3A* gene from the serotype 3 clinical isolate 0100993 [20] using the Janus cassette [25]. Aliquots of bacteria were stored at −70°C in THB containing 25% glycerol. For routine experiments, bacteria from frozen stocks were streaked onto THB agar containing 5% defibrinated horse blood and incubated for 12–24 h at 37°C with 5% CO_2_. Fresh colonies were scraped and transferred to THB and grew to desired density as measured by a spectrophotometer at OD 600_nm_.

### Synthetic CSPs

CSP1, CSP2 and their analogues (≥95% purity) were synthesized by Elim Biopharm (Hayward, CA).

### Activation assays of *comX* and *cbpD* promoters

The ability of synthetic CSPs to activate the promoters of *comX* gene was compared in D39pcomX::lacZ or TIGR4pcomX::lacZ bacteria grown in THB (pH 6.8) until OD 600_nm_ of 0.1, washed and resuspended in THB (pH 8.3). Induction of *cbpD* promoter was monitored using the D39pcbpD::lacZ cells grown under the same conditions. CSP1 or CSP2 and their analogues were added at indicated concentrations to the culture and incubated at 37°C for 30 min. ß-galactosidase activity was measured according to previously published protocols [19], and expressed as Miller units.

### *In vitro S. pneumoniae* transformation assay

*In vitro* transformation experiments were performed as we had previously described [20]. Briefly, *S. pneumoniae* cells were grown to early log phase (OD 600_nm_ 0.1) in THB (pH 6.8), washed and resuspended in THB (pH 8.3) containing *rpsL* DNA (1 µg). CSP1, CSP2 and their analogues were added alone or in combination at desirable concentrations. The *rpsL* gene, which confers resistance to streptomycin, and its flanking regions (combined length 1633 bp), were amplified from pneumococcal strain CP1296 [27] using the following primers: *rpsL* upper 5′-GGGCTAGTAGAAGTAGTTGG-3′; *rpsL* lower: 5′-CGGAAGTGTGCGAATGCACG-3′). The transformation mix was then incubated at 37°C with 5% CO_2_ for 1 hr. Transformants were selected on THB agar supplemented with 100 µg/mL streptomycin.

### Ethics statement

This study was carried out in strict accordance with the recommendations in the Guide for the Care and Use of Laboratory Animals of the National Institutes of Health. The protocol was approved by the Institutional Animal Care and Use Committee (IACUC) at the University of Illinois at Urbana-Champaign (Protocol Number: 10144).

### Mouse acute pneumonia infection assays

Mice used in this study were housed in positively ventilated microisolator cages with automatic recirculating water, located in a room with laminar, high efficiency particle accumulation–filtered air. The animals received autoclaved food, water, and bedding. Six-week old adult male CD-1 mice (Charles River, Boston) (groups of 10) were anesthetized with isoflurane and intranasally administered 3×10^6^ CFU of D39 bacteria or D39-20 µg CSP1-E1A mixture in 50 µl PBS. In a second experiment, CD-1 mice (groups of 20) were exposed to 2×10^6^ CFU of D39 bacteria or D39-100 µg CSP1-E1A mixture in 50 µl PBS. The Infected mice were monitored every 6 hr for 96 hr. Moribund animals that displayed rough hair coat, hunched posture, distended abdomen, lethargy or inability to eat or drink were euthanized.

### *In vivo* horizontal gene transfer studies

The *rpsL* donor DNA was amplified from pneumococcal strain CP1296 [25]. *S. pneumoniae* strain D39 cells were used as recipients. D39 cells were grown to OD 600_nm_ 0.1 in THB (pH 6.8), washed and resuspended in fresh THB (pH 8.3). Adult CD-1 mice were intranasally or intrapertoneally infected with D39 (3×10^7^, 50 µl volume) supplemented with a mixture of 100 ng CSP1 and 10 µg of *rpsL* gene DNA, or D39 supplemented with a mixture 100 ng CSP1, 800 ng CSP1-E1A, and 10 µg of *rpsL* gene DNA in THB. For natural transformation studies, a capsule-deficient *cap3A* mutant (Table 1) derived from a clinical serotype 3 strain 0100993 (20) was used as recipient. Natural transformation were performed intraperitoneally in mice with 2×10^9^ *cap3A* cells and 100 µg of chromosomal DNA from 0100993, with or without 100 µg of CSP1-E1A. No CSP1 peptide was used. For all transformation assays, individual reagents were kept separately on ice, mixed and immediately inoculated into animals to prevent the induction of competence prior to injection. Transformation event did not occur before or after the experiments because no transformants was recovered from plating the transformation mix before or after the experiments (data not shown). For intranasally infected mice, lungs were harvested 6hr post-infection, homogenized and plated on THB agar (total output) or THB agar supplemented with 100 µg/ml streptomycin for transformants. Intraperitoneally infected mice were monitored until they were moribund (∼16–24 hr) at which time the animals were euthanized, and the spleens were harvested for bacterial enumeration. The number of total bacteria and streptomycin resistant transformants in spleens were enumerated as described above.

#### *S. pnuemoniae* growth inhibition by CSP1 and derivatives

D39 cells were collected at early log phase (OD 600_nm_ 0.1). Cells (2×10^6^ cfu) were resuspended in 50 µl of sterile PBS and incubated with 20 µg or 100 µg of CSP1, CSP1-E1A or CSP1-R3A or CSP1, and incubated on ice for 10 min. THB (500 µl) was added to the cells/peptide mixtures, and incubated at 37°C in a CO_2_ incubator. Bacterial growth was monitored at OD 600_nm_ using a spectrophotometer for the indicated time intervals.

### Zymogram analysis of murein hydrolases

D39 cells were grown in THB (pH 6.8) to OD 600_nm_ of 0.1. Cells were washed and resuspended in fresh THB (pH 8.3). Cells were exposed to CSP1 (100 ng/ml) alone or to a combination of CSP1 and increasing amounts of CSP1-E1A (0, 100, 200, 400 or 800 ng/ml). After 30 minutes of incubation, cells were lysed by mixing with 2X SDS loading buffer, and subjected to zymogram analysis as previously published [60]. Lytic activity was observed as bands of clear zones in the turbid gel.

### Statistical analyses

Statistical analyses of *in vitro* experiments were performed using the Student's *t*-test and one-way analyses of variance (ANOVA). Statistical significance of survival studies was performed using the log-rank (Mantel-Cox) test in GraphPad Prism statistical software package. A significant difference was considered to be *p*<0.05.

### Accession numbers

gene = “comC1”, locus_tag = “SPD_2065”, db_xref = “GeneID:4441955”;

gene = “comD”, locus_tag = “SPD_2064”, db_xref = “GeneID:4442986”;

gene = “comE”, locus_tag = “SPD_2063”, db_xref = “GeneID:4441307”;

gene = “comX1”, locus_tag = “SPD_0014”, db_xref = “GeneID:4441078”;

gene = “comX2”, locus_tag = “SPD_1818”, db_xref = “GeneID:4442416”;

gene = “cbpD”, locus_tag = “SPD_2028”, db_xref = “GeneID:4442443”;

gene = “cap3A”, GenBank: Z47210.1.

## Supporting Information

Figure S1 **CSP1-E1A is not bacteriostatic against** ***S. pneumoniae*** **.** (A-C) D39 cells (2×10^6^ cfu) (OD 600_nm_ 0.1) were resuspended in 50 µl of PBS and incubated with 20 µg or 100 µg of CSP1, CSP1-E1A or CSP1-R3A or CSP1. After 10 min of incubation on ice, the cells/peptide mixture was added to 500 µl of THB, and incubated at 37°C. Bacterial growth was monitored by OD 600_nm_ for the indicated time intervals. Experiments were performed in triplicates and repeated three times. The means ± SD of one typical experiment are shown.
(TIF)
