# Supplementary figures and images for: Inhibition of Competence Development, Horizontal Gene Transfer and Virulence in Streptococcus pneumoniae by a Modified Competence Stimulating Peptide
Source: PLoS Pathog. 2011 Sep 1;7(9):e1002241. doi: 10.1371/journal.ppat.1002241 (PMC3164649; doi:10.1371/journal.ppat.1002241)

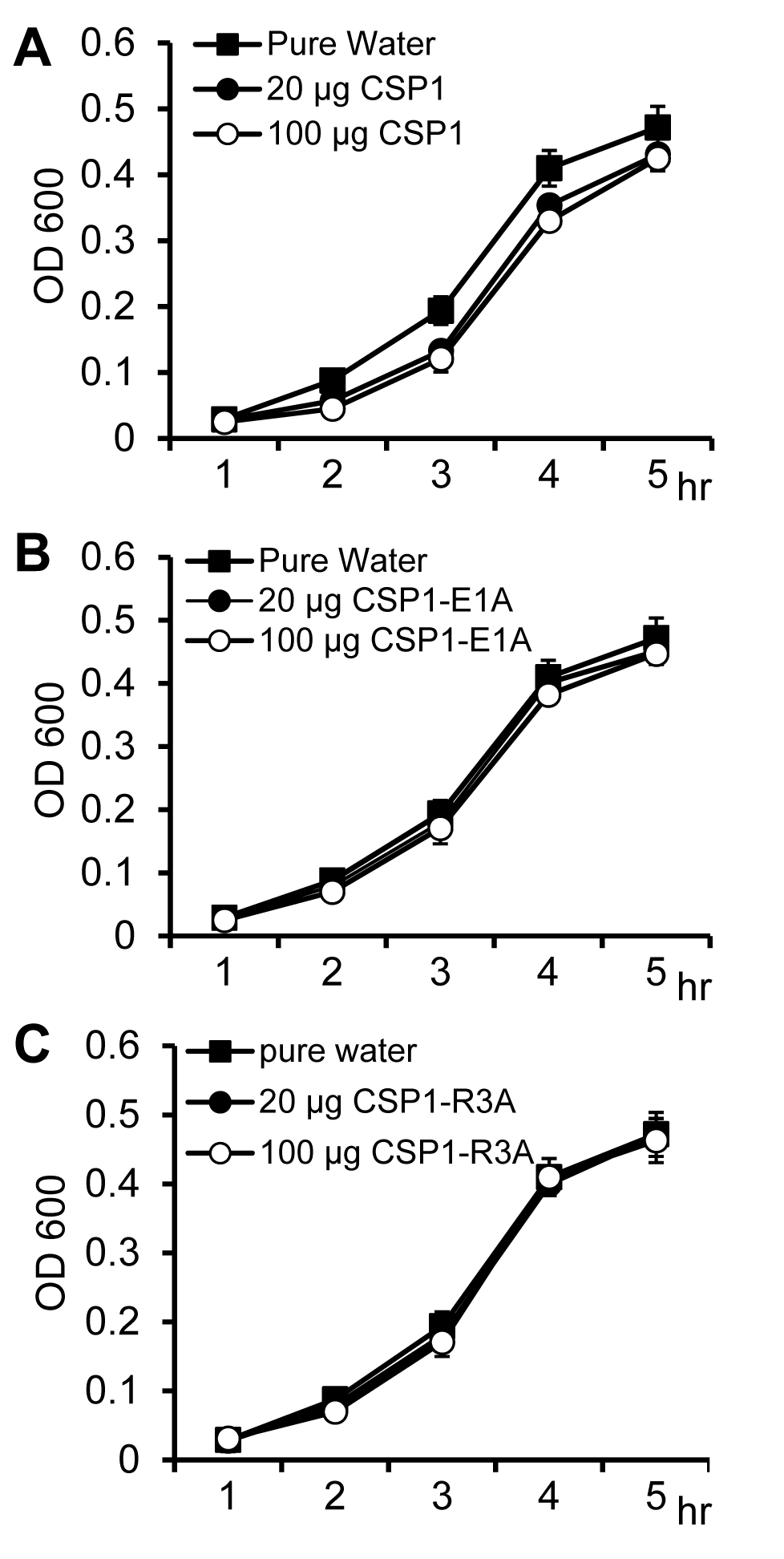

Supplement: Figure S1 — CSP1-E1A is not bacteriostatic against S. pneumoniae . (A-C) D39 cells (2×10^6^ cfu) (OD 600nm 0.1) were resuspended in 50 µl of PBS and incubated with 20 µg or 100 µg of CSP1, CSP1-E1A or CSP1-R3A or CSP1. After 10 min of incubation on ice, the cells/peptide mixture was added to 500 µl of THB, and incubated at 37°C. Bacterial growth was monitored by OD 600nm for the indicated time intervals. Experiments were performed in triplicates and repeated three times. The means ± SD of one typical experiment are shown. (TIF) [file ppat.1002241.s001.tif]
